# Supplementary material for: Active diffusion and advection in Drosophila oocytes result from the interplay of actin and microtubules
Source: Nat Commun. 2017 Nov 15;8:1520. doi: 10.1038/s41467-017-01414-6 (PMC5688094; doi:10.1038/s41467-017-01414-6)
Supplement: Supplementary file 1 — Supplementary Information [file 41467_2017_1414_MOESM1_ESM.pdf]

Supplementary Figures

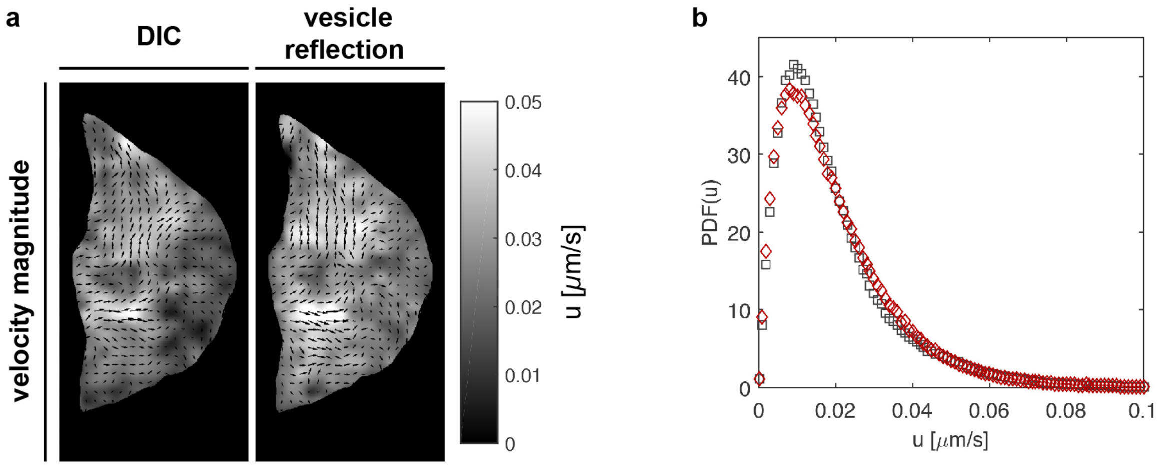

Supplementary Figure 1 (related to Figure 1) PIV analysis **(a)** Comparison between velocity fields (arrows) and speed distributions (intensity maps) obtained from PIV analysis on DIC and reflection microscopy images, respectively. Data shown are obtained from images taken simultaneously. Velocity fields and speed maps are shown after a boxcar average over a time window of 2.4 s. **(b)** Comparison between the speed probability distribution functions (PDF) obtained from PIV analysis on DIC (black squares, peak value: $(9\pm0.5)\cdot10^{-3}$ µm/s, mean value: $(20\pm1)\cdot10^{-3}$ µm/s) and reflection microscopy (red diamonds, peak value: $(9\pm0.5)\cdot10^{-3}$ µm/s, mean value: $(20.5\pm1)\cdot10^{-3}$ µm/s) image sequences for n=9 control cells.

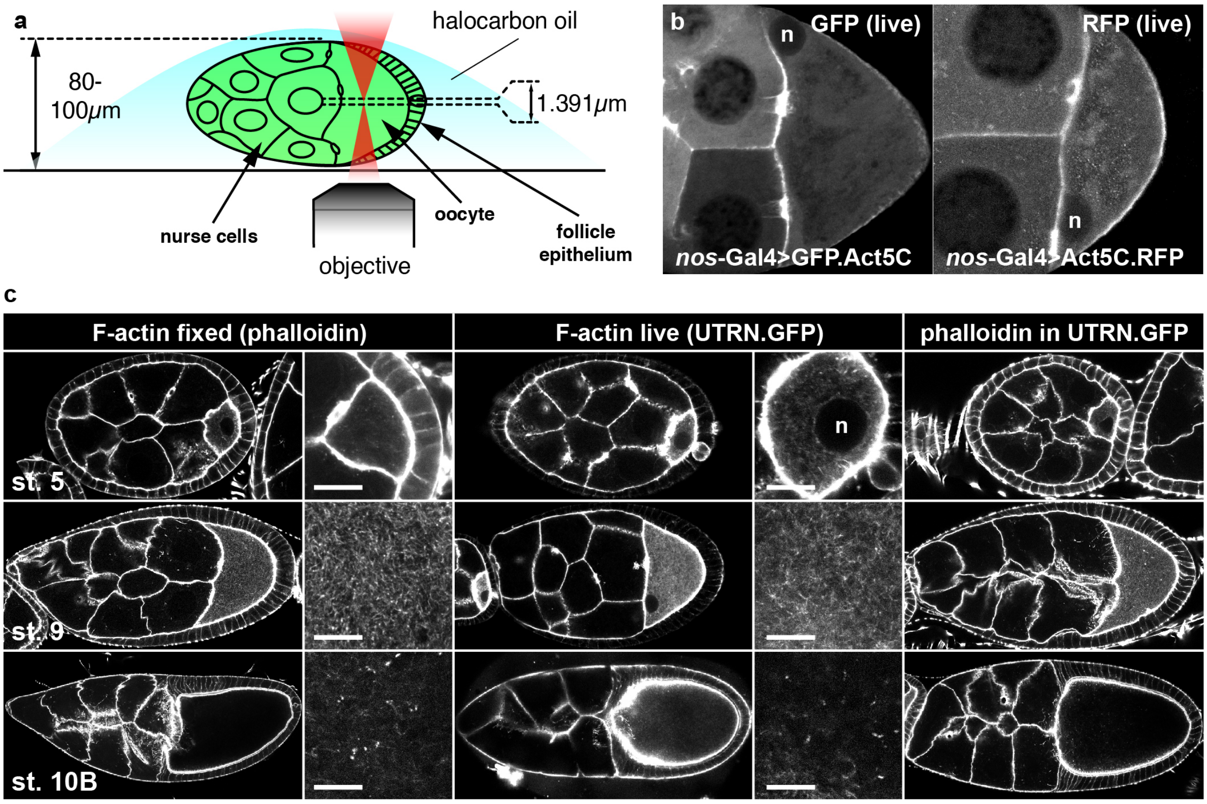

Supplementary Figure 2 (related to Figure 1 and 2) Imaging setup and F-actin labeling strategy in living cells **(a)** Schematic representation of the live imaging setup. Living egg chambers were dissected in halocarbon oil and imaged on an inverted confocal microscope. Confocal images (z=1.391μm) of fluorophore tagged proteins and vesicle reflection, as well as transmitted light images were acquired simultaneously. **(b)** Fluorophore tagged actin monomers driven by the germline specific driver *nanos*-Gal4 (*nos*-Gal4) become incorporated into the cortex of cells, but do not label cytoplasmic actin filaments. The position (N- or C-terminus) or the fluorophore (GFP or RFP) do not alter the behavior of the fluorophore tagged actin monomers. **(c)** UTRN.GFP constitutes an excellent tool to label cytoplasmic actin filaments, as the presence of UTRN.GFP does not alter the temporal regulation of mesh formation or dissolution. The mesh starts forming in young oocytes (st5) and becomes denser until st9. During st10 the mesh dissolves. This dynamic behavior of the cytoplasmic actin network is observed in fixed samples stained with TRITC-phalloidin (left panels), in living oocytes expressing UTRN.GFP (middle panels), and in fixed UTRN.GFP oocytes stained with TRITC-phalloidin (right panels). Scale bars are 10μm

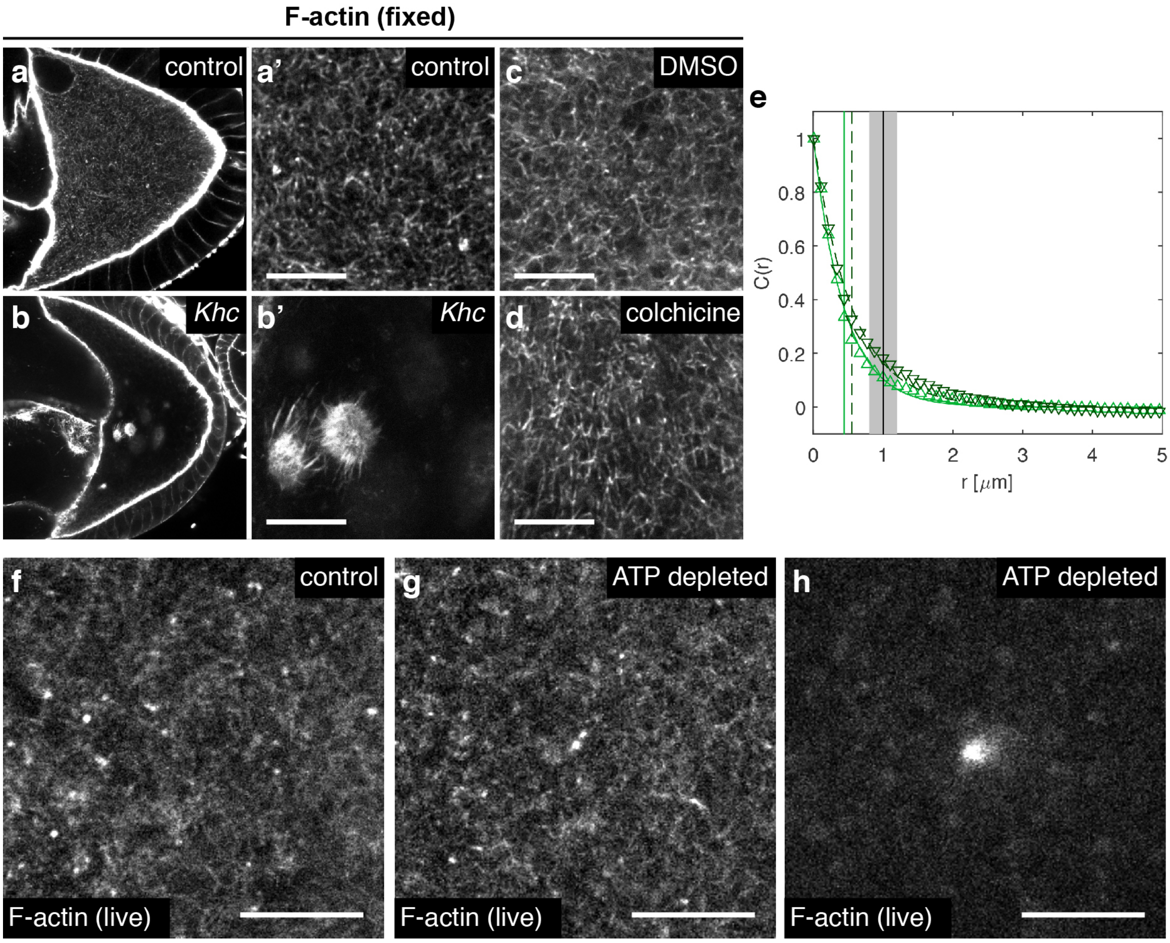

Supplementary Figure 3 (related to Figure 3): Morphology of the cytoplasmic actin network in oocytes without kinesin-1 or treated with colchicine or ATP depleting drugs **(a-b')** Control (a, a') or Kinesin-1 mutant (b, b') oocytes. Depletion of *Kinesin heavy chain (Khc)*, the catalytic subunit of kinesin-1, causes an aberrant accumulation of F-actin rich clusters in the cytoplasm (TRITC-phalloidin). **(c, d)** Compared to *Khc* mutants, the depolymerization of microtubules by colchichine, inhibiting all microtubule based transport and cytoplasmic flows, does not result in defects in the cytoplasmic actin network (TRITC-phalloidin). **(e)** Spatial intensity correlation functions of the actin mesh for control (upward green triangles) and colchicine-treated (downward dark green triangles) cells. Fitting the data to an exponential function allows to estimate the actin mesh correlation length: l_{act} = 0.42 ± 0.03 μm and l_{act} = 0.55 ± 0.05 μm, for control (continuous green vertical line) and colchicine treated (dashed dark green vertical line) cells, respectively. The dashed grey band corresponds to the average value ± standard deviation of the diameter of vesicles: d_{ves}=1.0 ± 0.2 μm. **(f-h)** Living UTRN.GFP expressing oocytes. As previously reported[1], long exposures of cells to ATP depleting drugs can cause the depolymerization of actin filaments (compare h to f). However, our acute treatment leaves the actin mesh intact in about half the cells investigated (g). For DDM analyses, only those cells were considered. Scale bars represent 10 *μ*m.

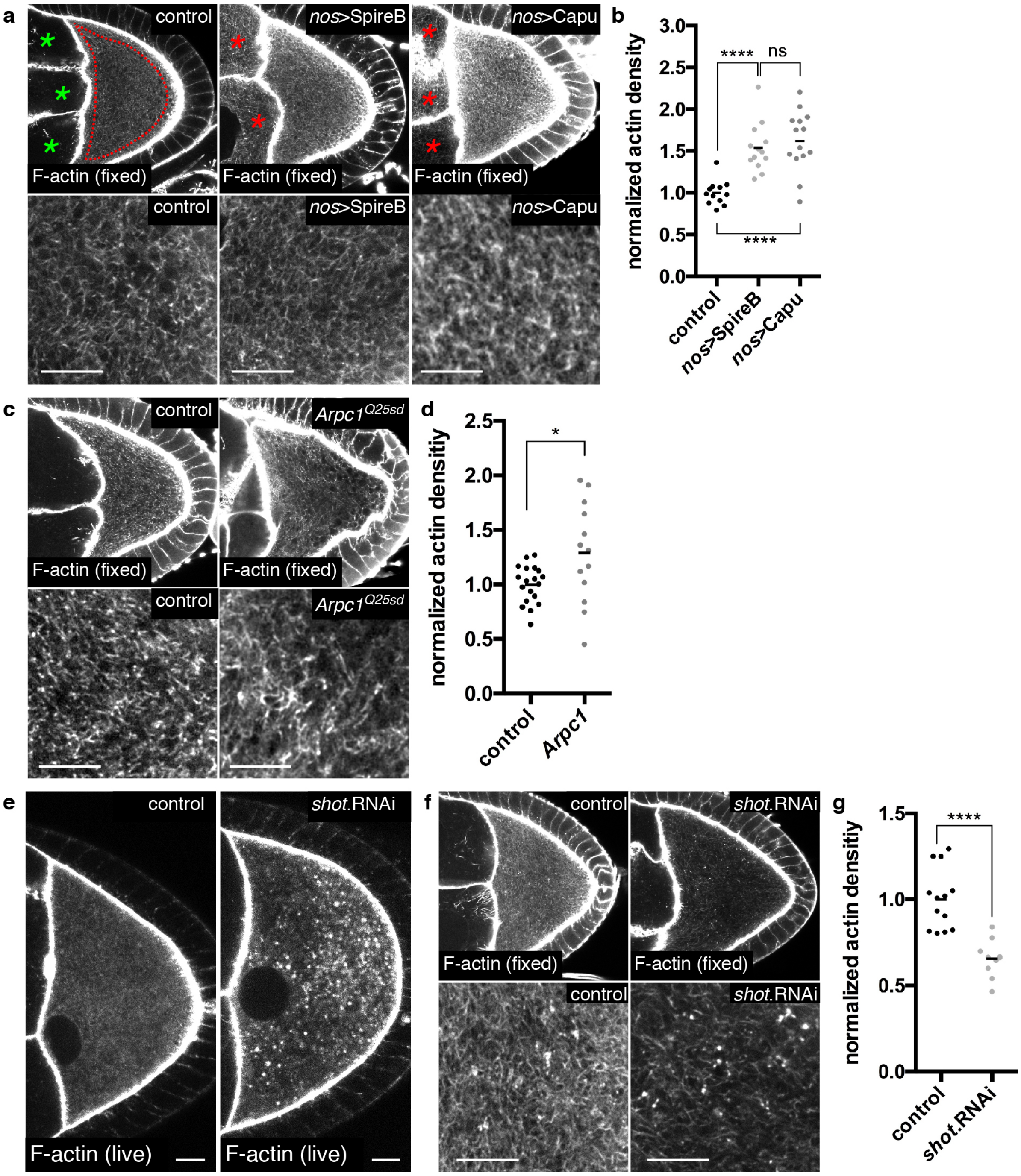

Supplementary Figure 4 (related to Figure 4) Effects of SpireB and Capu over-expression, mutation of Arpc1 and knock down of Shot on the density of F-actin in the oocyte **(a)** Fixed egg chambers stained with TRITC-phalloidin. Control egg chamber, expressing the germline specific driver *nos*-Gal4/+, and cells over-expressing SpireB or Capu by the same driver (*nos*>SpireB and *nos*>Capu) were fixed and stained simultaneously. During image acquisition, the over-expressing cells were identified by the presence of ectopic actin filaments in the nurse cells (compare green asterisks in *nos*-Gal4/+ with red asterisks in *nos*>SpireB or *nos*>Capu cells). Scale bars represent 10μm. **(b)** The apparent actin mesh density was estimated by measuring the mean intensities, normalized against the mean of the control cells, in single

oocytes. Each dot represents the normalized mean intensity of one cell. The measured area is outlined by a red dotted line in (b). Over-expression of SpireB or Capu leads to an increased intensity and therefore higher amount of F-actin within the cytoplasm. Data sets have been compared by an ANOVA test. **(c)** Impairment of the Arp2/3 actin nucleation complex (*Arpc1*^{Q25sd}) does not affect actin mesh formation, but overall actin organization in the cytoplasm. **(d)** Average F-actin levels are not dramatically changed in *Arpc1*^{Q25sd}, however a wider variation of mesh densities can be observed. Data sets have been compared by two-tailed, unpaired *t*-test. **(e)** UTRN.GFP in living control cells, or living cells in which *short stop* (*shot*) levels are reduced by RNAi. *shot*.RNAi cells present F-actin clusters in puncta throughout the cytoplasm. **(f)** In fixed *shot*.RNAi cells, a clear reduction of cytoplasmic actin filaments can be observed. **(g)** Normalized F-actin densities in control and *shot*.RNAi cells. Data sets have been compared by two-tailed, unpaired *t*-test. Scale bars represent 10μm.

Supplementary References

1 Bacallao, R., Garfinkel, A., Monke, S., Zampighi, G. & Mandel, L. J. ATP depletion: a novel method to study junctional properties in epithelial tissues. I. Rearrangement of the actin cytoskeleton. *J Cell Sci* **107 (Pt 12)**, 3301-3313 (1994).
