# Supplementary material for: Active diffusion and advection in Drosophila oocytes result from the interplay of actin and microtubules
Source: Nat Commun. 2017 Nov 15;8:1520. doi: 10.1038/s41467-017-01414-6 (PMC5688094; doi:10.1038/s41467-017-01414-6)
Supplement: Supplementary file 3 — Description of Additional Supplementary Files [file 41467_2017_1414_MOESM3_ESM.pdf]

Description of Additional Supplementary Files

File Name: Supplementary Movie 1
Description: Supplementary Movie 1 (related to Figure 1 and 2) Simultaneous imaging of F-actin (UTRN.GFP) and vesicles (DIC) in control cells. Images were acquired at 1 frame every 10.2s. Playback speed is 20 fps (~200x real speed).

File Name: Supplementary Movie 2
Description: Supplementary Movie 2 (related to Figure 3) Simultaneous imaging of F-actin (UTRN.GFP) and vesicles (DIC) in oocytes without microtubules. Images were acquired at 1 frame every 10.2s. Playback speed is 20 fps (~200x real speed).

File Name: Supplementary Movie 3
Description: Supplementary Movie 3 Imaging of mitochondria in the oocyte. Images were acquired at 1 frame every 1.3s. Playback speed is 80 fps (~80x real speed).

File Name: Supplementary Movie 4
Description: Supplementary Movie 4 (related to Figure 3) Simultaneous imaging of F-actin (UTRN.GFP) and vesicles (DIC) in ATP depleted cells. The observed overall movement results from a deformation of the cells during the imaging process, most likely due to weakened cortex and the absence of any ATPdependent forces. Images were acquired at 1 frame every 10.2s. Playback speed is 20 fps (~200x real speed).

File Name: Supplementary Movie 5
Description: Supplementary Movie 5 (related to Figure 4) Imaging of vesicles (DIC) under various conditions. Comparison of vesicle (DIC) movement in control cells (control), SpireB over-expressing cells (*nos*>SpireB), cells lacking the actin mesh (*spire* mutants) and cells lacking the actin mesh and microtubules (*spire* mutants + colchicine). Images were acquired at 1 frame every 1.3s. Playback speed is 80 fps (~80x real speed).
